# Trends in Diet Quality and Related Sociodemographic, Health, and Occupational Characteristics among Workers in Spain: Results from Three Consecutive National Health Surveys (2006–2017)

**DOI:** 10.3390/nu13020522

**Published:** 2021-02-05

**Authors:** Silvia Portero de la Cruz, Jesús Cebrino

**Affiliations:** 1 Department of Nursing, Pharmacology and Physiotherapy, Faculty of Medicine and Nursing, University of Córdoba, Avda. Menéndez Pidal S/N, 14071 Córdoba, Spain; n92pocrs@uco.es; 2 Department of Preventive Medicine and Public Health, Faculty of Medicine, University of Seville, Avda. Doctor Fedriani S/N, 41009 Seville, Spain

**Keywords:** diet, food consumption, occupational health, Spain, trends

## Abstract

Poor dietary practices are commonly reported in working populations from different economic sectors, resulting in increased absenteeism and a decrease in productivity. The aims of this study were to describe the frequency of food consumption and diet quality in workers aged ≥16 years from 2006 to 2017 in Spain and to evaluate the factors associated with diet quality. A nationwide cross-sectional study was carried out among workers using data from the Spanish National Health Surveys in 2006 (*n* = 11,068), 2011 (*n* = 7497) and 2017 (*n* = 8890). Sociodemographic, occupational, and health-related variables were used as well as diet quality data. A multiple linear regression was performed to determine the characteristics related to overall diet quality. The percentage of workers who consumed vegetables, at most, once or twice per week decreased from 2006 to 2017 (*p* < 0.001). A lower diet quality score was related to the consumption of tobacco and alcohol and being aged ≥25 years old, while a higher diet quality score was linked to being a woman, having Spanish nationality, receiving optimal perceived social support, being physically active in one’s main occupation, doing leisure-time physical activity, and the type of contract.

## 1. Introduction

Worldwide, having a poor diet is a major preventable cause of adverse health and many non-communicable diseases, such as diabetes, heart disease, stroke, and cancer [1,2,3]. Having an unhealthy diet is now generally considered a leading risk factor for disease and death [4]. Spain is a country with dietary recommendations based on the Mediterranean diet [5]. Nevertheless, the incidence of chronic conditions, such as cardiovascular, oncological, metabolic, and musculoskeletal diseases has risen in Spain over the last few decades, as has happened worldwide [6]. Although the Spanish dietary guidelines [7] promote healthy dietary patterns, a high percentage of individuals do not meet the existing dietary guidelines in terms of consumption of fruit and vegetables (94.6% consume less than the recommended five daily servings), cereals (84.2% consume less than four servings/day), whole grains (83.5% consume less than half a serving/day), and dairy (66.4% consume less than two servings/day) and consume higher quantities of meat than recommended (59.0% of subjects have more than one serving/day) [8]. Nowadays, the current dietary pattern is moving away from the traditional Mediterranean diet pattern towards increased consumption of energy-dense food characterized by poor nutritional quality [9].

Diet is associated with individual, lifestyle, social, economic, and geographical factors, amongst others [10,11,12,13], which can produce a social gradient in diet quality that contributes to health inequalities [14]. For instance, diet quality is positively related to increased age, higher educational level, engaging in physical activity, multivitamin use, and not living alone, and is negatively related to obesity and smoking [15,16,17,18].

Similar to the general population, risk factors for chronic conditions are prevalent in working individuals, specifically among those with low-income occupations [19]. Around the world, poor dietary practices have been reported in employed people from different economic sectors [20,21,22], resulting in absenteeism and productivity loss, which generate substantial costs for societies and employers [23,24]. To date, a number of workplace conditions, including work stress, long working hours, shift work, and the unavailability of foods to allow a healthy diet, have been reported as contributing factors to poor dietary practices [25,26]. However, the findings are varied and have been reported inconsistently, which may be explained, in part, by the limitations related to synthesizing both qualitative and quantitative studies. Given that a large number of adults spend lengthy periods at work, workplaces play an important role in providing an opportunity to inform workers about healthy eating [19].

To the best of our knowledge, this study is the first research study conducted in a large, representative working population that shows the evolution over time of eating habits and diet quality and also analyzes sociodemographic, occupational, and health-related factors as a prior step to developing and implementing sound healthy diet-promoting policies and practices in workplaces. This could play a key role in achieving the aims stated in Sustainable Development Goal 3.4 to reduce premature mortality from non-communicable diseases by one-third by 2030 [27]. The aims of the current study were to describe the frequency of food consumption and diet quality in workers aged ≥16 years from 2006 to 2017 in Spain and to evaluate the factors associated with diet quality.

## 2. Materials and Methods

### 2.1. Study Design, Data Source and Study Population

A nationwide, cross-sectional study was carried out. The sources of information were the Spanish National Health Survey (SNHS) 2006 [28], the SNHS 2011 [29], and the SNHS 2017 [30], all conducted by the Ministry of Health, Consumer Affairs and Social Welfare (MHCASW) and the National Institute of Statistics (NIS). The sampling framework consisted of non-institutionalized Spanish individuals. It used a three-stage sampling design. The first stage units were the census section, the second stage units were the households, and the third stage units were the individuals. The participants were informed about the survey through an informative letter from the MHCASW, detailing the purposes of the survey, the voluntary and anonymous nature of participation, and visit of a duly authorized interviewer.

The study population was restricted to individuals aged 16 to 64 years old who reported being in employment at the time that the surveys were conducted. The sample originally consisted of 31,501 subjects (SNSH 2006: *n* = 13,064; SNHS 2011: *n* = 8653; SNHS 2017: *n* = 9784). We excluded 4046 (12.84%) individuals who did not respond or refused to answer the interview questions (SNHS 2006: *n* = 1996; SNHS 2011: *n* = 1156; SNHS 2017: *n* = 894). Those individuals excluded were not different from the rest of workers. The total sample included 27,455 workers: 11,068 in SNHS 2006; 7497 in SNHS 2011; and 8890 in SNHS 2017.

### 2.2. Variables

Diet quality was the dependent variable, and it was examined through the Spanish Health Eating Index (SHEI) [31], which is a modified version of the North American Healthy Eating Index [32]. The SHEI is based on 10 food groups (cereals, vegetables, fruit, dairy, meat, legumes, cold meets, sweets, soft drinks and variety of the diet) classified into five categories (never or hardly ever, <1 time per week, 1–2 times per week, ≥3 times per week, but not daily, and daily), according to the frequency of food consumption proposed by the recommendations of the Spanish Society of Community Nutrition (SSCN) [33]. Each food group varies from 0 to 10 points, as reported by Supplementary Table S1. The total score for the SHEI was evaluated by summing the frequency of consumption of food groups. The higher the score, the higher the degree of compliance with the recommendations of SSCN. The overall SHEI score was categorized into three groups, taking into account the cut-offs previously described in the literature [32]: >80 points, good diet quality; 51–80 points, improvable diet quality; and <51 points, poor diet quality.

The independent variables were as follows:

Sociodemographic characteristics: survey year (2006, 2011, 2017); gender (woman, man); age (16–24 years, 25–44 years, 45–64 years); educational level (without studies, primary, secondary or professional training, university); marital status (single, married, widowed, separated/divorced); citizenship (Spanish, foreigner); and town size (<10,000 inhabitants, 10,000–100,000 inhabitants, >100,000 inhabitants).

Health-related factors: self-reported height and weight, which were used to calculate body mass index (BMI), which was classified as underweight (BMI below 18.50 kg/m^2^), normal weight (BMI of 18.50 to 24.99 kg/m^2^), overweight (BMI of 25.00 to 29.99 kg/m^2^), or obese (BMI of 30 kg/m^2^ or higher) [34], and common mental disorders, which were evaluated using the General Health Questionnaire (GHQ-12) [35], adapted to the Spanish language [36,37]. The questionnaire was composed of 12 items with four response options on a Likert scale from zero (more than usual) to three (much less than usual). The bimodal response scale was used. For this, if one of the first two options were answered, a score of zero was given and if one of the last two were answered, a score of one was given (0-0-1-1) [38]. The total score of the GHQ-12 was calculated by summing the 12 items (minimum score: 0 points and maximum: 12 points). The final score was categorized using a cut-off at ≥3 points as the absence of common mental disorders (<3 points) and presence of common mental disorders (≥3 points). Other variables in the study related to health were ongoing tobacco use (yes, no); alcohol consumption in the last 12 months (yes, no); physical activity in the main occupation (yes: walking, carrying some weight, frequently moving/carrying out tasks that require great physical effort, no: sitting down for most of the day/standing up for most of the day, without moving much or making an effort); and leisure-time physical activity (yes, no).

Perceived personal social support: this was evaluated using the Duke-UNC-11 questionnaire [39], which was validated in Spain [40,41]. The questionnaire consists of 11 items evaluated using a scale of five possible responses, ranging from one (Much less than I would like) to five (As much as I would like). The total score is calculated as the sum of scores for all items. Social support varies from 11 to 55 points. The level of social support was categorized into optimal support (≥33 points) or sub-optimal support (≤32 points).

Occupational variables: the company activity in which the adult works (primary economic activity, secondary economic activity, tertiary economic activity). This activity was categorized according to the National Classification of Economic Activities 1993 [42] and 2009 [43]. Other occupational variables used in the study were type of contract (without contract, self-employed, temporary contract, ongoing contract, civil servant or permanent contract); type of working day (regular: split working day and continuous morning shift; irregular: continuous afternoon shift, continuous night shift, shift work, reduced working day and irregular working day); work-related stress (evaluated by a Likert scale ranging from one “not at all stressful” to seven “very stressful”, categorizing as: none or low work-related stress (1 and 2 points), moderate work-related stress (3–5 points) and high work-related stress (6 and 7 points)); and job satisfaction (evaluated by a Likert-type response format ranging from one “not satisfactory” to seven “very satisfying”, grouped as no or low job satisfaction (1 and 2 points), moderate job satisfaction (3–5 points) and high job satisfaction (6 and 7)).

### 2.3. Procedure and Ethical Aspects

Anonymized data, which are available to the public, were downloaded from the NIS and MHCASW websites [29,30,31]. According to Spanish laws, Ethics Committee approval is not necessary if secondary data are being used. The Supplementary File contains the research data.

### 2.4. Statistical Analysis

The workers are described through the counts and percentages for categorial variables and arithmetic means and standard deviations (SDs) for quantitative variables. For the bivariate analysis, the 2-means Student’s *t*-test was employed, while for the analysis of three or more means, the ANOVA repeated means test was applied. Linear regression models were performed to recognize significant trends in the frequency of food consumption from 2006 to 2017 using the regression coefficient and the coefficient of determination (R^2^). In addition, a multiple linear regression (forward stepwise selection) was made using diet quality as the result variable. Previously, variables relevant to the final model were selected from the univariate analysis (variables with *p* < 0.15 were removed from the model). All possible interactions were assessed. Variables with *p* > 0.05 were assessed as potential confounding factors and were considered as such if the percentage of change in the coefficient was >20%. To assess goodness of fit, the adjusted coefficient of determination (R^2^), the F statistic and the normality of the residues were analyzed. Values of *p* < 0.05 were considered significant. For statistical analysis, the IBM SPSS Statistics version 25 (IBM Corp, Armonk, NY, USA), licensed to the University of Cordoba (Spain) was used.

## 3. Results

### 3.1. Sociodemographic, Occupational and Health-Related Variables

A total of 27,455 workers constituted the total sample, including 47.54% (*n* = 13,052) women and 52.46% (*n* = 14,403) men with a mean age of 42.50 years (SD ± 10.15). Most of them were married (60.08%), lived in towns with >100,000 inhabitants (41.30%), had completed secondary-level study (57.29%), were Spanish (91.70%), were of normal weight (48.58%), were non-smokers at the time of study (66.22%), had consumed alcohol in the past twelve months (68.47%), were not physically active at work (77.27%) but were active during their leisure time (60.27%), had no common mental disorders (85.37%), and received normal social support (97.33%). Regarding occupational variables, most of the participants had a current contract (51.81%), had an irregular working day (59.94%), belonged to the tertiary sector of the economy (71.38%), had moderate levels of perceived stress (58.82%), and had a high level of job satisfaction (51.00%).

### 3.2. Food Consumption and Diet Quality

**Figure 1:**
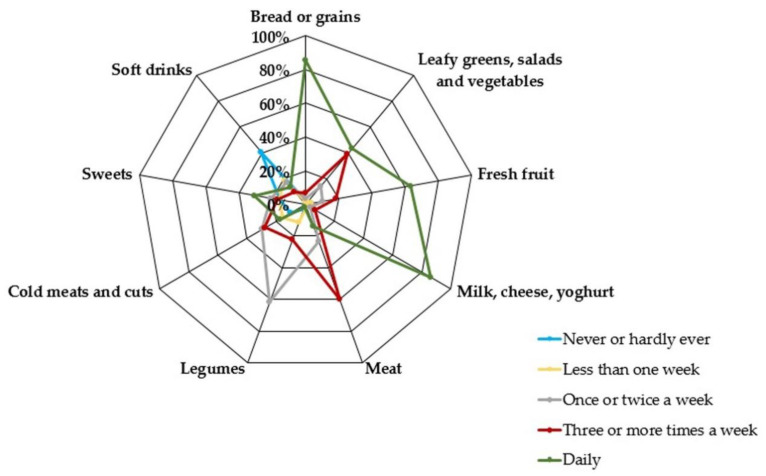
Frequency of food consumption in workers aged 16–64 years (*n* = 27,455) (2006–2017).

Most of the participants reported daily consumption of bread or grains (85.69%), fresh fruit (63.41%), and milk, cheese, or yoghurt (86.19%). In addition, 30.88% consumed sweet foods daily (Figure 1). Regarding diet quality, this needed to be improved in 70.89% of the subjects.

From 2006 to 2017, the percentage of workers who consumed leafy greens, salads, and vegetables, at most, once or twice per week decreased (2006: 23.70%, 2011: 17.28%, 2017: 11.43% (B = −0.78, R^2^ = 0.95, *p* < 0.001)). On the other hand, there was an increase in the consumption of milk, cheese, or yoghurt ≥3 times per week but not in daily consumption (2006: 4.18%, 2011: 7.08%, 2017: 9.79% (B = 10.15, R^2^ = 0.99, *p* = 0.04)). In addition, there was an increase in workers who consumed soft drinks ≥3 times per week but not in daily consumption (2006: 9.59%, 2011: 9.94%, 2017: 10.48% (B = 1.53, R^2^ = 0.98, *p* = 0.04)) (Table 1).

### 3.3. Association between Sociodemographic, Occupational, and Health-Related Variables and Diet Quality

The bivariate analysis revealed no relationship between a poor diet quality and marital status, size of town of residence, BMI, common mental disorders, or work-related stress. In the adjusted multivariate analysis, workers who were over 25 years old, had a temporary contract, and consumed tobacco and alcohol had lower mean diet quality scores. In contrast, there were positive significant relationships between diet quality and being female, being Spanish, being physically active in one’s main occupation, doing physical activity during leisure time, perceiving optimal social support, being self-employed, and being a civil servant or having a permanent contract or being a non-contracted worker (Table 2).

## 4. Discussion

The main results of this study suggest that the frequency of food consumption did not vary among workers from 2006 to 2017, except for the consumption of milk, cheese and yoghurt, leafy green, salads, vegetables, and soft drinks. Similarly, the overall quality of the workers’ diets did not vary over the years of the study. On the other hand, the quality of diet was related to gender, age, nationality, type of contract, consumption of tobacco and alcohol, physical activity, and social support.

Spanish dietary recommendations are based on the traditional Mediterranean diet [44]. In our study, most of the respondents from the Spanish working population consumed bread (85.69%), fruit (63.41%), vegetables (43.29%), and milk (86.19%) daily. In addition, 61.64% of the workers in our study consumed legumes once or twice per week, while the frequency of consumption was lower for meat (23.49%). Although the Mediterranean dietary pattern is also dominant in countries such as Croatia and Portugal [45,46], a study carried out in workers from Croatia [47] showed higher bread consumption (91%) and lower consumption of fruit (51.20%) and vegetables (33.60%) than in our study. In Portugal, the frequency of consumption of fruit and vegetables was found to be higher (53% and 76%, respectively), but it was lower for milk (74.60%) and meat (17%) compared with the results of our study [48]. Paradoxically, and despite the fact that the Nordic diet has many similarities regarding the use of fruit and vegetables in the Mediterranean diet [49], Hemiö et al. [50] reported a lower intake of fruit and vegetables among Finnish workers (27.97% and 22.97%, respectively) than in the workers in the current study. While the Brazilian diet is based on the consumption of high-fat foods, salt, and sugars [51], the frequency of legume consumption in Brazilian workers was found to be similar to that found in our study (62.60%) [52]. In addition, the findings of the present study corroborate the results of a previous study, indicating that one-third of workers consumed sweet foods daily [53]. It is important to consider that a comparison between countries needs to be considered carefully, owing the use of different dietary guidelines and survey methods to assess the frequency of food consumption.

In the current study, diet needed to be improved in 70.89% of the workers. Diet quality is determined by a multitude of individual, social, and organizational factors. A number of studies have reported several barriers to maintaining a healthy diet for workers including long working hours, shift work and high workloads, low staffing levels, moderate levels of self-efficacy, and a lack of nutritional knowledge [25,54]. In contrast, the availability of healthy food options, the enjoyment of healthy behaviors, and social support have been reported as facilitators of eating healthy [55].

The percentage of workers who consumed leafy greens, salads, and vegetables, at most, once or twice per week decreased from 2006 to 2017, and those who consumed milk, cheese, or yogurt ≥3 times per week, but not daily, increased in our study. Workplaces are potentially promising environments for health promotion, due to the fact that workers spend one-third of their time there [56]. In Spain, health promotion strategies in the workplace are a relatively recent phenomenon. The Spanish Strategy on Occupational Safety and Health (SSOSH) 2007–2012 [57] and 2015–2020 [58] aimed to promote the improvement of health and safety conditions at work through companies adopting health promotion plans, which include activities aimed at following a healthy diet, preventing a sedentary lifestyle or avoiding unsuitable work postures [57,58]. In contrast, in the present study, the percentage of workers who consumed soft drinks at least once per week—but not daily—increased from 2006 to 2017. A study conducted in Nepal reported that the main barriers to healthy eating, as informed by the working population, are the unavailability of healthy food, the lack of sufficient human resources to prepare more healthy food options, and the greater cost of healthy foods [59].

When analyzing the predictors of diet quality in our study, women were found to have an increased diet quality score compared with men. Similar results have been obtained in other studies [15,60,61], and this could be explained by the fact that women are more health-conscious than men, and they may be more worried about the quality of their foods as well as having different job roles or shorter working hours, which may allow female workers to have more control over dietary choices [60,62,63]. However, in another study, no associations were found between those variables [64]. Although older age has been related to higher diet quality [65], in the current study, older workers had lower mean diet quality scores. Possible reasons for this finding could be that appetite and sensory abilities diminish with age [66]. Moreover, medical conditions, the use of drugs, and dental changes, affecting mouth movement and chewing ability, usually increase with age and could affect eating behavior, food preparation, and food intake [67].

Our results also corroborated previous findings [14,68], indicating that, in the univariate analysis, workers with a higher level of education had an increased overall diet quality score compared with employees with no education. This is probably due to people with a higher level of education having greater knowledge about nutrition, better cooking skills, or the ability to follow prevention messages. Regarding nationality, we found that Spanish employees’ dietary quality was better than that of foreign workers. This finding is consistent with another study conducted in Canada [69]. Immigrant individuals generally show lower rates of healthy behavior than the majority of non-immigrant people. Factors contributing to suboptimal eating behavior among immigrant groups include abnormally high social, economic, and environmental vulnerability due to the impacts of acculturation, low health literacy, and other migration-specific effects on health [70]. Moreover, it has also been suggested that the community-based participatory research approach has led to sustained improvements in the dietary quality of the foreign population [70]. In line with our results, most studies have shown that social support is usually associated with better diet quality [71,72,73]. It should be noted that social support may be a significant resilience resource, as individuals may rely upon their work colleagues, family, or friends [74].

There has been little research into the impact on diet quality of different health-related behaviors, such as smoking and alcohol consumption, or that of physical activity in the working population [75,76,77]. Regarding tobacco consumption, we found that smokers had lower diet quality scores. This result is similar to those obtained in studies conducted in Luxembourg [78], China [79], United States [80], and Australia [16]. Generally, smokers often have higher intakes of energy, total fat, saturated fat, cholesterol, and alcohol and lower intakes of antioxidant vitamins, fruit, and vegetables, in comparison with non-smokers [78]. It has been estimated that 32.10% of Spanish workers smoke [81]. This consumption is influenced by occupational conditions such as shift work, long working hours, high job demands, and a low degree of autonomy [82,83]. In a working population, alcohol consumption may be influenced by occupational factors and is strongly correlated with the particular worksite and the time of shift [84]. In the present study, employees who consumed alcohol presented a worse diet quality due, in part, to the higher energy intakes attributable to alcoholic beverages. A recent systematic review [85] showed that even alcoholic beverage preferences are associated with particular dietary patterns, according to the area of residence. In Western countries, and to a lesser extent in Mediterranean countries, people with a preference for wine have, in general, healthier dietary habits than individuals with other preferences. Consistent with previous studies [76,86], work-related physical activity and leisure-time physical activity were found to be related to better diet quality. Eating behavior and physical activity both use the same decision-making process, which involves self-regulation mediated by the neural system [87]. However, occupational and leisure-time physical activity have different characteristics. For example, in occupational physical activity, there is often little control over the number of tasks and speed at which the employees perform these activities. On the contrary, leisure-time physical activity is directly under one’s control and is usually performed in short bouts with breaks [88]. Feig et al. [89] showed that, although undergoing more work-related physical activity is not related to a healthier diet, employees’ leisure-time physical activity was closely linked to a healthier dietary intake, which suggests that physical activity carried out on the job may not be a strong indication of a healthy diet per se.

Regarding job characteristics, our results tie in with previous evidence [90] pointing to a significant link between the type of contract and the quality of diet. Thus, we found that diet quality scores were lower for temporary workers than that for their colleagues with ongoing contracts. The relationship between temporary work and health is complex, although it has been explained by unhealthy behavior in response to job insecurity and uncertainty about the terms and conditions of the job [91,92,93]. In contrast, civil servants were more likely to follow a healthy diet, reinforcing the potential role of favorable working conditions in adopting a healthy dietary pattern [94]. One interesting finding in our study was that the worse the working conditions for the self-employed and non-contracted workers were, the higher the diet quality scores were. A possible explanation for this could be that those employees have more support from family networks, which can act as a buffer against the negative dietary consequences of poor working conditions [95]. In the current study, the univariate analysis showed that working in the secondary economic sector with the tertiary sector decreased diet quality scores compared. Currently, 18.7% of the Spanish workforce are employed in the secondary economy sector [96]. It has been reported that employees in that sector consume fewer fruit and vegetables and may also be less likely to participate in worker wellness programs. This highlights the need to implement health promotion strategies that motive workers to make positive choices regarding leading a healthy lifestyle [97]. In contrast, working in the primary sector increased the diet quality score compared with working in the tertiary sector. In the context of agriculture, for instance, it has been stated that workers on farm-household systems which are more commercialized and better connected to markets tend to have more healthy diets than those who work for subsistence-oriented households [98].

Regarding the type of working day, employees with a regular working day had greater diet quality scores in the univariate analysis. This finding has been reported previously [75]. Higher consumption of saturated fats and soft drinks has been identified among employees with an irregular working day [99]. Moreover, taking into account that employees with an irregular working day are exposed to chronobiological and hormonal changes as a consequence of their working hours, they could present metabolic alterations due to excessive consumption of these foods [100,101]. Moreover, it should be pointed out that the diet quality scores were higher among employees with a high level of job satisfaction than for those who had a low level. Job satisfaction is considered the most important factor in the overall life satisfaction of employees, surpassing satisfaction with health and social life [102]. A prior study reported that life satisfaction is positively related to healthy food choices [103]. Additionally, some authors have suggested that having a positive mood may stimulate food intake, resulting in larger servings on the plate, which leads, in turn, to overeating and excessive calorie intake and a downward spiral toward obesity [104,105].

### 4.1. Strengths and Limitations

This study and its results had certain limitations. First, due to the cross-sectional design, it was not possible to establish cause and effect relationships. Second, the use of the SHEI to evaluate the diet quality provided the frequency of consumption by food groups rather than by quantities of food or energy. However, the validity of the Health Eating Index (HEI) has been demonstrated in studies with plasma biomarkers [106,107], where a high HEI score was associated with blood concentrations of particular markers with a protective effect against certain diseases. Third, BMI was calculated from the heights and weights reported by the participants, which may not be accurate. Fourth, the significant differences obtained for the frequency of food consumption over the study period should be considered with caution, given the small number of workers with respect to the total number at each time point. Finally, the analyzed data came from three surveys conducted on different samples. Despite the fact that the survey was the same, differences in the characteristics of the sample may have influenced the results. Nonetheless, one strength of the current study is that the data were obtained from national representative surveys, contributing to a gain in knowledge on this topic for today’s society.

### 4.2. Implications for Research and Practice

In light of our findings, comprehensive effort is needed to devise relevant approaches to improve workers’ nutrition across the sociodemographic gradients of diet quality and, thus, to avoid the growing burden of diet-related ill health. Moreover, further longitudinal research conducted in the workplace is required to evaluate the roles of work-related characteristics on the diet quality of employed people. The results of the current study can be used to guide and inform the improvement of health promotion guidelines and polices in the workplace. Future workplace dietary interventions should be focused on promoting interventions that can effectively prevent and reduce workers’ unhealthy diets, taking into account psychological and environmental factors. Moreover, it is essential to plan further evaluations of diet quality over time to track changes related to disease and to monitor the adherence of the workers to the dietary guidelines and recommendations.

## 5. Conclusions

The study found that the number of workers consuming milk, cheese, or yoghurt and soft drinks three or more times per week, but not daily, from 2006 to 2017, increased, while, the number of workers consuming leafy greens, salads, and vegetables, at most, once or twice per week fell. From 2006 to 2017, the quality of the workers’ diets, in general, did not vary. Diet quality scores were lower in workers who were over 25 years old, had a temporary contract, or who consumed tobacco and alcohol, and higher in those who were female, Spanish, physically active in their main occupation, who did physical activity during their leisure time, perceived that they had an optimal social support, or were self-employed, civil servants, on a permanent contract, or were non-contracted employees.

## Figures and Tables

**Table 1:** Frequency of food consumption in workers aged 16–64 years (*N* = 27,455) (2006–2017).

Variables	2006	2011	2017	*B*	R^2^	*p*-Value
	*n* = 11,068 (%)	*n* = 7497 (%)	*n* = 8890 (%)			
Frequency of consumption of bread or grains						
Never or hardly ever/<1 time per week/1–2 times per week	751 (6.79%)	632 (8.43%)	631 (7.10%)	1.59	0.45	0.52
≥3 times per week ^1^	526 (4.75%)	629 (8.39%)	761 (8.56%)	6.68	0.74	0.34
Daily	9791 (88.46%)	6236 (83.18%)	7498 (84.34%)	−8.01	0.49	0.5
Frequency of consumption of leafy greens, salads and vegetables						
Never or hardly ever/<1 time per week/1–2 times per week	2622 (23.70%)	1296 (17.28%)	1017 (11.43%)	−0.78	0.95	<0.001
≥3 times per week ^1^	3806 (34.39%)	2611 (34.83%)	4217 (47.44%)	24.08	0.82	0.28
Daily	4640 (41.92%)	3590 (47.89%)	3656 (41.12%)	−2.6	0.03	0.9
Frequency of fresh fruit (excluding juices) consumption						
Never or hardly ever/<1 time per week/1–2 times per week	2191 (19.80%)	1448 (19.31%)	1267 (14.25%)	−1.07	0.83	0.27
≥3 times per week ^1^	1648 (14.89%)	1472 (19.63%)	2020 (22.72%)	13.99	0.97	0.11
Daily	7229 (65.31%)	4577 (61.05%)	56.03 (63.03%)	−4.44	0.24	0.67
Frequency of consumption of dairy products (milk, cheese, yoghurt)						
Never or hardly ever/<1 time per week/1–2 times per week	596 (5.38%)	565 (7.54%)	766 (8.62%)	1.16	0.95	0.15
≥3 times per week ^1^	463 (4.18%)	531 (7.08%)	870 (9.79%)	10.15	0.99	0.04
Daily	10,009 (90.43%)	6401 (85.38%)	7254 (81.60%)	−16.89	0.98	0.09
Frequency of meat (chicken, beef, pork, lamb, etc.) consumption						
Never or hardly ever/<1 time per week/1–2 times per week	2651 (23.95%)	2276 (30.36%)	2304 (25.92%)	0.36	0.52	0.49
≥3 times per week ^1^	6317 (57.07%)	4444 (59.28%)	5682 (63.91%)	12.00	0.98	0.09
Daily	2100 (18.97%)	777 (10.36%)	904 (10.17%)	−15.71	0.72	0.35
Frequency of legume consumption						
Never or hardly ever/<1 time per week/1–2 times per week	8225 (74.31%)	5848 (78.00%)	6761 (76.05%)	1.27	0.18	0.72
≥3 times per week ^1^	2500 (22.59%)	1563 (20.85%)	2069 (23.27%)	1.25	0.1	0.79
Daily	343 (3.10%)	86 (1.15%)	60 (0.67%)	−4.37	0.86	0.25
Frequency of consumption of cold meats and cuts						
Never or hardly ever/<1 time per week/1–2 times per week	6240 (56.38%)	4523 (60.33%)	4354 (48.98%)	-0.51	0.92	0.18
≥3 times per week ^1^	2575 (23.27%)	1793 (23.92%)	3183 (35.80%)	23.19	0.83	0.27
Daily	2253 (20.36%)	1181 (15.75%)	1353 (15.22%)	-9.31	0.78	0.31
Frequency of consumption of sweets (biscuits, pastries, jams, cereals with sugar, sweets, etc.)						
Never or hardly ever/<1 time per week/1–2 times per week	5730 (51.77%)	4141 (55.24%)	4493 (50.54%)	−0.86	0.24	0.67
≥3 times per week ^1^	1414 (12.78%)	1111 (14.82%)	2087 (23.48%)	19.71	0.92	0.19
Daily	3924 (35.45%)	2245 (29.95%)	2310 (25.98%)	−17.49	0.98	0.09
Frequency of consumption of soft drinks with sugar						
Never or hardly ever/<1 time per week/1–2 times per week	8125 (73.41%)	5835 (77.83%)	7110 (79.98%)	0.31	0.66	0.4
≥3 times per week ^1^	1061 (9.59%)	745 (9.94%)	932 (10.48%)	1.53	0.98	0.04
Daily	1882 (17.00%)	917 (12.23%)	848 (9.54%)	−13.61	0.96	0.13
Diet quality						
Poor diet quality	520 (4.70%)	143 (1.91%)	204 (2.29%)	−4.25	0.58	0.45
Improvable diet quality	8008 (72.35%)	4616 (61.57%)	6800 (76.49%)	8.27	0.1	0.79
Good diet quality	2540 (22.95%)	2738 (36.52%)	1886 (21.21%)	−5.03	0.02	0.9

B = Estimated parameter; R^2^ = Adjusted coefficient of determination; ^1^ But not daily.

**Table 2:** Univariate and adjusted linear regression models predicting diet quality scores in workers aged 16–64 years (*n* = 27,455) (2006–2017).

Variables	Mean (SD)	Univariate Analysis			Adjusted Analysis		
		B	*β*	*p*-Value	B	*β*	*p*-Value
Gender							
Man	71.48 (11.02)	Reference	Reference		Reference	Reference	
Woman	74.57 (10.65)	3.09	0.14	<0.001	2.96	0.14	<0.001
Age group (years)							
16–24	64.05 (12.21)	Reference	Reference		Reference	Reference	
25–44	56.84 (10.94)	−7.21	−0.33	<0.001	−6.27	−0.29	<0.001
45–64	52.02 (9.77)	−12.03	−0.54	<0.001	−10.71	−0.48	<0.001
Educational level							
No education	72.82 (11.30)	Reference	Reference				
Primary	72.37 (11.32)	−0.45	−0.01	0.35			
Secondary or professional training	72.19 (11.15)	−0.63	−0.03	0.16			
University	74.86 (10.04)	2.04	0.08	<0.001			
Marital status							
Single	70.24 (11.61)	Reference	Reference				
Married	74.07 (10.43)	3.83	0.17	0.07			
Widowed	76.62 (9.89)	6.38	0.07	0.15			
Separated/Divorced	73.91 (10.63)	3.67	0.09	0.23			
Citizenship							
Foreigner	69.82 (11.15)	Reference	Reference		Reference	Reference	
Spanish	70.06 (10.89)	0.24	0.09	<0.001	1.56	0.04	<0.001
Town size							
<10,000 inhabitants	73.05 (10.69)	Reference	Reference				
10,000–100,000 inhabitants	71.77 (11.00)	−1.28	−0.11	0.09			
>100,000 inhabitants	73.36 (11.05)	0.31	0.01	0.19			
Body Mass Index							
Normal weight	69.35 (11.85)	Reference	Reference				
Underweight	65.71 (11.05)	−3.64	−0.04	0.59			
Overweight	69.28 (10.67)	−0.07	−0.003	0.83			
Obese	69.31 (11.15)	−0.04	−0.001	0.38			
Ongoing tobacco use							
No	74.18 (10.46)	Reference	Reference		Reference	Reference	
Yes	70.51 (11.48)	−3.67	−0.16	<0.001	−2.62	−0.11	<0.001
Alcohol consumption in the last12 months							
No	73.56 (11.17)	Reference	Reference		Reference	Reference	
Yes	72.67 (10.84)	−0.89	−0.04	<0.001	−5.17	−0.02	<0.001
Physical activity as main activity							
No	70.95 (11.26)	Reference	Reference		Reference	Reference	
Yes	73.54 (10.79)	2.59	0.09	<0.001	1.17	0.05	<0.001
Leisure-time physical activity							
No	71.23 (11.25)	Reference	Reference		Reference	Reference	
Yes	74.07 (10.60)	2.84	0.13	<0.001	2.39	0.11	<0.001
Perceived social support							
Sub-optimal social support	70.36 (11.94)	Reference	Reference		Reference	Reference	
Optimal social support	73.02 (10.91)	2.66	0.04	<0.001	2.03	0.03	<0.001
Common mental disorders							
Absence of common mental disorders	72.71 (11.71)	Reference	Reference				
Presence of common mental disorders	72.99 (10.82)	0.28	0.009	0.14			
Sector of economy							
Tertiary	73.59 (10.84)	Reference	Reference				
Secondary	72.15 (10.63)	−1.44	−0.6	<0.001			
Primary	74.55 (11.13)	0.96	0.04	<0.01			
Type of contract							
Ongoing contract	72.90 (10.84)	Reference	Reference		Reference	Reference	
Without contract	76.84 (10.67)	3.94	0.06	<0.001	1.12	0.02	0.01
Self-employed	75.09 (9.80)	2.19	0.08	<0.001	0.76	0.03	<0.01
Temporary contract	67.13 (11.45)	−5.77	−0.2	<0.001	−2.3	−0.08	<0.001
Civil servant or permanent contract	76.03 (12.10)	3.13	0.14	<0.001	1.35	0.06	<0.001
Type of working day							
Irregular	72.65 (11.08)	Reference	Reference				
Regular	73.38 (10.74)	0.73	0.03	<0.001			
Work-related stress							
None or low	73.05 (11.27)	Reference	Reference				
Moderate	73.02 (10.79)	−0.03	−0.001	0.87			
High	72.70 (11.12)	−0.35	−0.01	0.1			
Job satisfaction							
None or low	71.40 (11.73)	Reference	Reference				
Moderate	72.36 (10.80)	0.96	0.04	0.36			
High	73.59 (10.96)	2.19	0.1	<0.01			

SD = Standard Deviation; B = Estimated parameter; β = Beta coefficient; Adjusted coefficient of determination (R^2^) = 13.50%, F = 30.61, *p* = < 0.001.

## Data Availability

The data presented in this study are available as Supplementary Material (File S1: Research data).

## Supplementary Materials

The following are available online at https://www.mdpi.com/2072-6643/13/2/522/s1, Table S1: Criteria to define the score for each item in the Spanish Health Eating Index (SHEI), File S1: Research data.
