# Supplementary material for: Trends in Diet Quality and Related Sociodemographic, Health, and Occupational Characteristics among Workers in Spain: Results from Three Consecutive National Health Surveys (2006–2017)
Source: Nutrients. 2021 Feb 5;13(2):522. doi: 10.3390/nu13020522 (PMC7915096; doi:10.3390/nu13020522)
Supplement: Supplementary file 1 [file nutrients-13-00522-s001.zip › Supplementary Table S1.docx]

**Supplementary Table S1.** Criteria to define the score for each item in the Spanish Health Eating Index (SHEI).

| **Criteria to define the score for each item in the Spanish Health Eating Index (SHEI)** | | | | | |
| --- | --- | --- | --- | --- | --- |
| **Variables** | Criteria for a maximum score of 10 | Criteria for a maximum score of 7.5 | Criteria for a maximum score of 5 | Criteria for a maximum score of 2.5 | Criteria for a maximum score of 0 |
| Daily |  |  |  |  |  |
| Bread or grains | Daily | Three or more times per week, but not daily | Once or twice per week | Less than once per week | Never or almost never |
| Leafy greens, salads and vegetables | Daily | Three or more times per week, but not daily | Once or twice per week | Less than once per week | Never or almost never |
| Fresh fruit (excluding juices) | Daily | Three or more times per week, but not daily | Once or twice per week | Less than once per week | Never or almost never |
| Dairy products (milk, cheese, yoghurt) | Daily | Three or more times per week, but not daily | Once or twice per week | Less than once per week | Never or almost never |
| Weekly consumption |  |  |  |  |  |
| Meat (chicken, beef, pork, lamb, etc.) | Once or twice per week | Three or more times per week, but not daily | Less than once per week | Daily | Never or almost never |
| Legumes | Once or twice per week | Three or more times per week, but not daily | Less than once per week | Daily | Never or almost never |
| Occasional consumption |  |  |  |  |  |
| Cold meats and cuts | Never or almost never | Less than once per week | Once or twice per week | Three or more times per week, but not daily | Daily |
| Sweets (biscuits, pastries, jams, cereals with sugar, sweets, etc.) | Never or almost never | Less than once per week | Once or twice per week | Three or more times per week, but not daily | Daily |
| Soft drinks with sugar | Never or almost never | Less than once per week | Once or twice per week | Three or more times per week, but not daily | Daily |
| Variety | 2 points if participant achieve each of the daily recommendations, 1 point if participant achieve each of the weekly recommendations. | | | | |

Each item scored from 0 to 10 depending to the criteria of the Spanish Health Eating Index (SHEI) [1], where 10 points means that the recommendations proposed by the Spanish Society of Community Nutrition (SSCN) are fulfilled [2].

Supplementary references

1. Norte Navarro, A.; Ortiz Moncada, R. Spanish diet quality according to the healthy eating index. *Nutr*. *Hosp*. **2011**, *26*, 330–336, doi: 10.1590/S0212-16112011000200014.
2. Spanish Society of Community Nutrition (SSCN). Healthy dietary guidelines (2014). Available online: <http://www.nutricioncomunitaria.org/es/otras-publicaciones> (accessed on 13 December 2020).
